# Bibliometric analysis of the Brazilian contribution to anesthesiology and pain management publications

**DOI:** 10.1590/1806-9282.20250021

**Published:** 2025-07-07

**Authors:** Italo Carvalho Martins, Luís Phelipe Gama de Moraes, Bianca Sousa Belfort Ferreira, Ian Gabriel Lucchese de Sá Cruz, Vinicius Freire Pereira, Isabela Pinheiro Souza, Ed Carlos Rey Moura, Caio Márcio Barros de Oliveira, Plínio da Cunha Leal

**Affiliations:** 1 Universidade Federal do Maranhão, Department of Medicine – São Luís (MA), Brazil.

## INTRODUCTION

Anesthesiology and pain management are becoming ­increasingly vital in enhancing both perioperative care and chronic pain treatment, which has sparked a notable increase in global research efforts. To get a clearer picture of the evolving trends, key contributors, and emerging topics in these fields, ­researchers often rely on bibliometric analysis, a method that quantitatively assesses academic literature^1^. Over the past decade, research in anesthesiology has experienced significant growth, especially in perioperative pain management. This surge is largely driven by initiatives such as Enhanced Recovery After Surgery (ERAS) protocols and the adoption of multi-modal pain relief strategies^2-4^.

Countries like the United States, the United Kingdom, and Germany have led the way in advancing this research, with many of their contributions featured in high-impact journals and reflected in top-cited studies^3^. A recent bibliometric study focused on perioperative analgesia, which highlighted the ­growing academic focus on improving surgical outcomes through better pain management techniques, has also called attention to the significant contributions from prominent institutions and global leaders in the field^4^.

Despite the overall momentum in anesthesiology research, there is a noticeable lack of focus on Brazil's role in these advancements. While Brazil is recognized as a key player in public health research across Latin America^5^, its contributions to anesthesiology research have not been extensively explored, with no major bibliometric studies addressing this gap. This presents a valuable opportunity for further investigation. The aim of this bibliometric analysis is to evaluate the contributions of Brazilian researchers to the fields of anesthesiology and pain management, examining trends in publication, international collaboration, and the broader impact of their work. By assessing Brazil's position in the global research community, this study seeks to identify opportunities to enhance Brazil's research contributions and encourage more international partnerships in anesthesiology and pain management.

## METHODS

A comprehensive bibliometric search of publications in anesthesiology and pain medicine from Brazil was conducted using the Scopus database provided by Elsevier. This analysis did not have temporal limitations and focused on the top 10 journals with the highest CiteScore in these fields. The selected journals included *Anaesthesia, British Journal of Anaesthesia*, *Pain, Journal of Headache and Pain* (open access), *Anesthesiology, Anesthesia and Analgesia*, *Best Practice and Research in Clinical Anaesthesiology, Seminars in Arthritis and Rheumatism*, *Journal of Pain and Symptom Management*, and *Canadian Journal of Anesthesia*. The search, carried out on September 16, 2024, was refined by applying filters based on the publication location (Brazil) and journal selection criteria.

The bibliometric data from these 10 journals were exported in BibTeX format and subsequently analyzed using R Studio software (version 4.2.3, R Foundation for Statistical Computing, Vienna, Austria). The analysis was performed with the "­bibliometrix" package, a robust tool for the quantitative assessment of ­scientific publications. To visually represent the findings, Google Sheets was used to create graphs, enhancing the ­visualization of ­publication volume, collaborative efforts, and the overall scientific impact of Brazilian contributions to these fields.

## RESULTS

According to data on anesthesiology publications, Brazil has 280 published articles during this period, with 198 conducted through national collaborations, *single-country publications* (*SCPs*), and 82 through multinational collaborations, *multiple-country publications* (*MCPs*), representing 29.3% of the total. The United States ranks first in collaboration with Brazil, contributing 55 articles. European countries such as Germany and France also make significant contributions, with 20 and 16 articles, respectively. These data highlight the strong involvement of developed countries in international collaborations.

**Figure 1:**
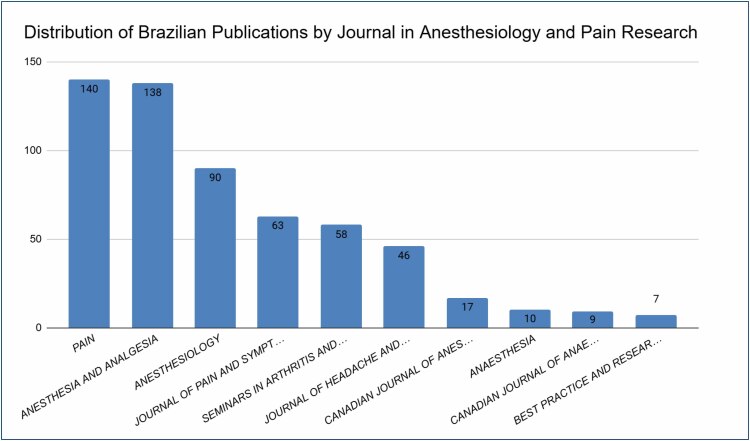
Number of articles published per journal. Legend: Bar chart showing the number of Brazilian publications in anesthesiology and pain by journal. Taller bars represent journals with higher publication volume.

The bibliometric analysis of Brazilian contributions to anesthesiology and pain management publications reveals ­significant insights into the sources of these articles. As illustrated in Figure 1, the number of articles published across ­various sources varies widely.

The journal *Pain* leads with the highest number of publications, totaling 140 articles. This is closely followed by *Anesthesia and Analgesia* with 138 articles. *Anesthesiology* also shows a substantial contribution with 90 articles. Other notable sources include the *Journal of Pain and Symptom Management* and *Seminars in Arthritis and Rheumatism*, each contributing between 58 and 63 articles. *The Canadian Journal of Anesthesia* has published a total of 26 articles. *Best Practice and Research: Clinical Anaesthesiology* has the least number of articles, with only seven publications.

**Figure 2:**
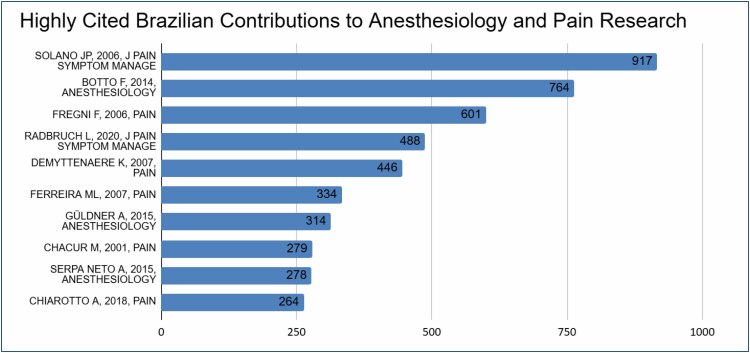
Total citations per paper.

The graph in Figure 2 illustrates the number of citations received by various research articles. The x-axis shows the total number of citations, while the y-axis lists the articles themselves. The article Solano JP (2006, J PAIN) holds the highest number of citations, reaching 900. After that, Botto F (2014) has around 750 citations, and Fregni F (2006, PAIN) has gathered around 600 citations.

Other articles with notable citation counts include Radbruch L (2020, J PAIN) with around 500 citations and Demyttenaere K (2007) with around 400 citations. Ferreira ML (2007, PAIN) received close to 300 citations, while articles by Güldner A (2015), Chacur M (2001, PAIN), Serpa Neto A (2015), and Chiarotto A (2018, PAIN) have between 250 and 200 citations.

These data reveal the significant academic impact of these articles, with citation counts serving as an indicator of their relevance and influence within the field.

**Figure 3:**
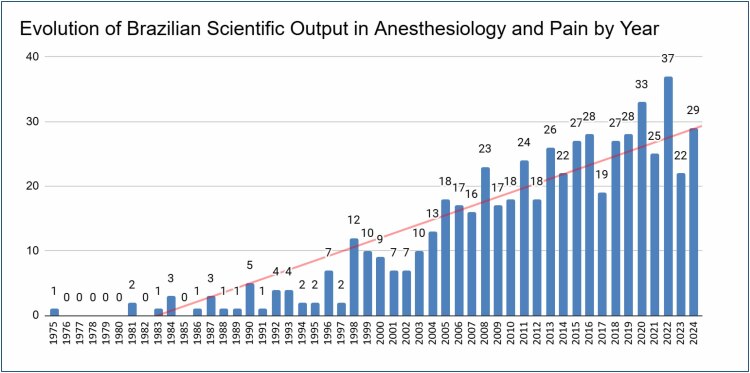
Articles published per year.

The analysis of Brazilian contributions to anesthesiology and pain management publications from 1980 to 2023 shows a notable increase in the number of articles published over the years. As depicted in Figure 3, there has been a steady rise in the total number of publications, with significant fluctuations during certain periods.

The red line in the graph represents a trend line, indicating the cumulative number of publications, which has shown consistent growth, especially after the year 2000. This suggests a growing interest and investment in research within these fields. The blue line, representing the annual number of publications, displays more variability, with several peaks and troughs. These variations could be due to factors, such as changes in research funding, the introduction of new technologies, or shifts in academic focus.

## DISCUSSION

The bibliometric analysis of Brazilian publications in anesthesiology and pain management reveals important aspects of the impact and relevance of the country's scientific contribution to these fields. One of the main points observed was the profile of international collaborations. Brazil predominantly ­cooperates with countries in North America and Europe, particularly with the United States. Global studies show similar behavior, in which the United States also stands out as the main collaborator in various areas of anesthesiology^6^.

This pattern of collaboration reflects the global trend toward partnerships with countries with greater infrastructure and research capacity, which favors the dissemination and impact of publications. Nonetheless, there is a gap in collaborations with countries in Asia and Africa. Expanding partnerships with these continents could enhance the diversity and reach of Brazilian research, as collaborations with emerging countries are considered a strategic opportunity to advance investigations in neuromodulation, for instance^7^.

Another aspect observed by the analysis is the distribution of publications in high-impact scientific journals. The journals *Pain* and *Anesthesia and Analgesia* lead the number of Brazilian publications, with 140 and 138 articles, respectively, followed by *Anesthesiology* and the *Journal of Pain*, which also make a substantial contribution, with 90 and 58 articles published, respectively. These journals are widely recognized for their high impact factor, making them central platforms for the dissemination of research in the fields of anesthesiology and pain management.

It can be seen that the international visibility and number of citations of a piece of research are directly related to the choice of journals with a higher impact factor^8^. Thus, the preference of Brazilian researchers for these journals is strategic, aiming to maximize the reach of their work while ensuring the dissemination of their findings in major journals.

An analysis of the productivity of Brazilian authors in anesthesiology and pain management publications showed a greater concentration of publications in a small group of researchers, namely "Pelosi P," with 32 publications, followed by "Rocco PRM," with 20 publications, showing that a significant part of scientific production in the area is restricted to a few renowned authors. This phenomenon is common in many scientific fields and reflects the specialization and continuity of long-term research projects^9^. Comparative studies in other fields, such as pain management in children, show similar patterns, where a few authors dominate academic production, indicating their leadership and expertise in their respective areas^6^.

With regard to the years of publication in Brazil, there has been a steady growth in Brazilian scientific production in anesthesiology and pain management, especially since the 2000s, with visible evolution. However, it can be seen in detail that the sharp angular coefficient of the graph reflects a rhythm of growth marked by oscillations, which could be smoothed out by investing in research in proportion to regional needs. Up until the 2000s, according to the study's data, the number of publications was no more than 10 per year.

This scenario changed in the following decades, illustrated by the increase in the number of Brazilian members of the International Association for the Study of Pain (IASP), reaching 4th place worldwide, with 2,813 members, behind only the United States, China, and Germany. The increase in financial resources, infrastructure, laboratory practices, and incentive programs aimed specifically at anesthesiology has boosted the development of new projects and the training of more researchers^10^. This scenario accelerated Brazil's scientific production in this field and increased its international ­visibility over the decades.

### Limitations

This study has some limitations. First, the analysis was conducted exclusively using data from the Scopus platform, which may have restricted the scope of the results. Including other databases, such as PubMed and Embase, would have enhanced the depth and reliability of the analysis. Additionally, the temporal limitation of the data used may have excluded recent studies that either have not been published yet or have not accumulated a significant number of citations, potentially affecting the representativeness of the findings.

## CONCLUSION

The bibliometric analysis demonstrates the growing impact of Brazilian publications in anesthesiology and pain management, driven by increased visibility in high-impact journals. Although collaborations are primarily with North America and Europe, expanding partnerships to Asia and Africa could further diversify the research. The concentration of publications among a few specialized authors indicates the need for greater researcher participation to sustain growth. Consistent investments in research and infrastructure are crucial, and including more databases in future analyses could improve the representativeness of the results. In conclusion, Brazil plays a central role in advancing global knowledge and improving patient care in these fields.

## Data Availability

The datasets generated and/or analyzed during the current study are available from the corresponding author upon reasonable request.
